# Exploring the determinants of online health service usage intentions under the threat of air pollution

**DOI:** 10.3389/fpubh.2024.1447733

**Published:** 2024-10-02

**Authors:** Xueru Xu, Tao Wang, Chuang Liu, Li Luo, Xiaofei Liu

**Affiliations:** ^1^West China Hospital of Sichuan University, Chengdu, China; ^2^Business School, Sichuan University, Chengdu, China; ^3^School of Finance and Business, Chengdu Vocational & Technical College of Industry, Chengdu, China

**Keywords:** air pollution, online health services, protection motivation, rational choice, behavioral intention

## Abstract

**Introduction:**

The rapid evolution of online health services (OHS) has significantly improved healthcare accessibility. However, the impact of air pollution on individuals’ willingness to engage with OHS remains underexplored in the existing literature. This study addresses this gap by investigating how air pollution threats influence the intention to use OHS, grounded in Protection Motivation Theory (PMT) and Rational Choice Theory (RCT).

**Methods:**

Using cross-sectional survey data, a theoretical model was developed and tested to examine the relationships between threat appraisal, coping appraisal, perceived benefits, perceived costs, and OHS usage intentions.

**Results:**

The findings indicate that threat appraisal, coping appraisal, and perceived benefits positively affect the intention to use OHS, while perceived costs negatively impact usage intention. Additionally, age significantly moderates the relationship between coping appraisal and the intention to use OHS.

**Discussion:**

This study extends the application of PMT and RCT in the context of OHS. It provides valuable insights into the factors influencing users’ intentions to utilize these services, with implications for improving OHS adoption in environments affected by air pollution.

## 1 Introduction

In the era of digital medical care, the online health services (OHS) is an innovative product to address the problems of imbalances in medical resources, high costs of accessing medical services, and other medical challenges (1). It utilizes the internet platform to offer comprehensive medical services, including consultation, diagnosis, treatment, and education. This provides patients with convenient and efficient medical care, as well as access to a wider range of medical resources (2, 3). OHS has become a mainstream trend in the field of healthcare, with platforms like Med Help, Good Doctor Online, Best Doctor, Zocdoc, and WellDoc gaining increasing popularity (4). According to a report in *Science*, in early of 2020, the use of Mayo Clinic’s digital medical services increased by 10,880% (5). In China, as of December 2022, the number of OHS users has reached 363 million. OHS has been widely applied in various healthcare scenarios, including psychiatry, dermatology, and emergency departments (6–8). However, some studies have indicated that not all individuals are willing to embrace or hold a positive attitude towards OHS (9, 10).

To effectively manage OHS demands and enhance product upgrades, both industry and academia are committed to understanding the patterns and behaviors of OHS users’ needs, with a particular focus on the factors influencing users’ willingness to use OHS. Personal characteristics, policies, medical resources, and technological convenience are the primary factors discussed. However, OHS essentially serves as an alternative channel to offline clinics, as it shares many of the same functions. Therefore, the factors influencing customers’ use of OHS are more complex. In addition to personal characteristics and resource levels, external environmental factors also play a role. Moreover, the extent of influence from environmental factors varies depending on the individual’s level of awareness. To more comprehensively analyze the willingness to use OHS, this study will discuss the impact of air pollution, an environmental factor to which people are highly sensitive (11), on the willingness to use OHS. Additionally, it discusses whether the extent of the impact of air pollution is influenced by individual characteristics.

In existing studies, patient characteristics (i.e., age, sex, socioeconomic status, cultural acceptance, and eHealth literacy) (12), policy (i.e., restrictions on disease types and the medical insurance reimbursement policy) (13), medical resource factor (i.e., hospital size, hospital grade, and physician competency) (14), and technology factor (i.e., the complexity of system operation, the completeness of system functions, and the degree of information interoperability) (15, 16) are the primary influencing factors. That research provides valuable knowledge as the basis for understanding OHS customer intention, but little attention has been paid to consumers’ usage of OHS from the environment perspective. Existing studies have confirmed that air pollution not only affects individuals’ subjective emotions and life satisfaction (17, 18), but also influences behavior. For example, in the field of economics and marketing management research, there is strong evidence that air pollution has negative impact on customers purchase behavior (19, 20), leading them to choose online alternative services to avoid the harm caused by air pollution. A large body of research in the domain of healthcare revealing the harm of air pollution to individual health (21, 22) and changes in health management plans (23). In practical terms, as an important alternative to offline healthcare, OHS has the capability to significantly reduce patients’ exposure to outdoor air pollution by eliminating the necessity for travel. If individuals perceive a high threat potential from air pollution (threat appraisal), individual may stay in home and use OHS. Therefore, our paper addresses the following research question:

RQ1: Does the threat of air pollution influence consumers’ intentions to use OHS?

Furthermore, when individuals consider the efficacy of protective actions against air pollution, their intentions to adopt the recommended behavior (e.g., use OHS) may be strengthened. Thus, individuals’ coping appraisal of adoption OHS is another key factor. Protection motivation theory (PMT), which explains why individuals engage in protective behaviors in response to perceived health threats (24), serves as the theoretical foundation of this study. However, older adult individuals may exhibit differences in their coping appraisal when it comes to OHS, particularly due to the IT components of OHS. Consequently, similar to internet products, the OHS acceptance level among the older adult is relatively low (25). As Wilson et al. observed, older adult individuals face barriers due to a lack of self-efficacy, knowledge, support, functionality, and information about the benefits of e-health for older adults, when use e-health (26). Accordingly, we propose the second research question:

RQ2: Does age moderate the relationship between the assessment of coping appraisal with OHS and under the threat of air pollution?

Additionally, individuals’ assessments of the cost and benefit of OHS are another important factor influencing their willingness to use it. Rational choice theory (RCT) (27) can helps us understand how the perception of OHS cost and benefit influences customers’ willingness. Thus, we combine the PMT and RCT to proposes a theoretical model that assess how air pollution, as an external threat factor, influences patients’ willingness to use OHS. We collected 542 questionnaires online and used structural equation modeling (SEM) to validate our theoretical model. The findings of this study have demonstrated that threat appraisal, coping appraisal, perceived cost, and perceived utility all impact the willingness to use OHS. Furthermore, age moderates the relationship between coping appraisal and OHS usage intention. Our study provides valuable insights into users’ intentions to utilize OHS.

This research contributes significantly to the existing body of knowledge. Firstly, this study fills gaps in the literature by exploring how air pollution influences patients’ willingness to use OHS. Secondly, by integrating PMT and RCT, it provides a comprehensive analysis of the determinants of OHS usage under environmental health threats. Lastly, the study reveals that age moderates the impact of coping appraisal on OHS adoption, with the positive effect diminishing as age increases. Based on our findings, our study offers two practical implications. First, it provides guidance for healthcare service providers to understand demand fluctuations during periods of air pollution, enabling them to timely adjust the allocation of online and offline resources. Second, we identified the heterogeneity in OHS adoption among different age groups, which can inform the adaptation and upgrading of OHS products to better cater to the needs of various age demographics.

Following this introduction, section 2 presents the prior research about the relationship between air pollution and human behavior, and the background of basic theoretical. Section 3 demonstrates our research model and hypotheses development, and then section 4 demonstrates methods. Section 5 discusses the research results and, at the end, section 6 summarizes our findings and gives implications for future research.

## 2 Literature review

### 2.1 Air pollution and human behavior

Air pollution is listed as the fourth highest risk factor affecting human health in the world today (28). Substances such as photochemical oxidants (smog), carbon monoxide, nitrogen oxides, sulfur oxides, and particulate matter in the air cause respiratory, cardiovascular, and immune system diseases, seriously affecting human health (29, 30). According to the “Urban Air Quality Database” released by the World Health Organization, only 12% of the global population lives in cities with air quality that meets the standard (31). Air pollution is a significant environmental issue that poses a variety of health risks and 7 million people die each year from diseases related to air pollution (32).

In addition to affecting physical health, exposure to air pollution can also impact neurological function and human social-neuro behavioral responses (33, 34), which is also a key focus of this study. The existing studies believing that there is a negative correlation between air pollution and consumer decision-making behavior (35). For example, there is a negative correlation between the severity of air pollution in tourist destinations and tourists’ outdoor activities, itineraries, and inbound travel behavior (36, 37). Besides, Wang et al. (38) found that as the air quality of the departure location deteriorates, the demand for outbound tourism increases, indicating a driving effect of air pollution. Those conclusions have been validated in 99 countries (39). Similarly, He et al. (19) confirmed that deteriorating air quality reduces cinema attendance, even for major films, leading to box office losses. In terms of perception, air pollution harms people’s happiness and satisfaction, as well as mental health (40). Based on review of relevant literature (41), it can be concluded that when consumers face high pollution environments, they tend to reduce outdoor activities, take protective measures (such as increasing health-related expenditures), and even move to cities with better air quality.

In the field of healthcare research, the majority of studies concentrate on exploring the intricate relationship between air pollution and various health metrics, such as morbidity (24, 42), mortality (43, 44), and hospitalization rates (45). However, there remains a paucity of research delving into behavioral modifications triggered by air pollution. Some studies have discussed the causative effects of air pollution triggering people’s short-term avoidance behaviors, including the masks adoption, utilization of air purifiers, intake of medication (46), and demand for health insurance (47). Based on these findings, it can be surmised that when individuals perceive the negative impacts of air pollution, they will take action to avoid it. Nevertheless, it is currently unknown whether air pollution can stimulate more OHS activities. During the pandemic, an increasing number of people have turned to online shopping and food delivery, which indicates a shift in consumer habits from contact consumption to contactless consumption (48). We believe that a similar phenomenon may emerge in health management. Therefore, this article discusses the inherent relationship between air pollution and OHS activities. In the OHS scenario, services can be provided at the same level as offline clinics while reducing exposure to external pollution. Consequently, we believe that air pollution affects the intention to use OHS. However, there is a lack of research on this factor, necessitating further investigation.

Additionally, discussing the impact of air pollution on consumer decision-making at the group level is a valuable direction for future research. When exploring the impact of air pollution on home-buying behavior, studies have found that people who have previously lived in highly polluted areas are more sensitive to air pollution (49). Research (50) also indicates that air pollution affects audit quality, and the characteristics of auditors mediate the relationship between air pollution and audit quality. In healthcare settings, when faced with high pollution, healthcare management consumption behavior is moderated by income levels (51, 52). Through a review of literature related to OHS behaviors, we found that age is one of the key factors influencing customer’s behavior (25, 26). The main reason is that older individuals have lower acceptance and usage capabilities for IT technology (53, 54). Therefore, this study considers age as an important moderating variable to analyze the behavioral differences between younger and older customers.

### 2.2 Rational choice theory

The theory of rational choice provides a framework for understanding how individuals make decisions and act between multiple options. It posits that, in decision-making situations, individuals systematically evaluate the significance and worth of various alternatives to arrive at the most optimal choice. This model encompasses the following elements: firstly, the individuals involved and the set of alternative options available; secondly, the individual’s comprehension of all pertinent values linked to each potential option; thirdly, the comparability of values or preferences across options; and finally, the individuals’ pursuit of maximizing their utility or value function through informed choice (27). Consequently, the theory of rational choice offers a powerful tool for explaining various events, actions, social processes, or institutional frameworks (55).

Practitioners generally acknowledge that the theory of rational consumption can describe what research subjects should do and predict their actual actions. This theory originally found its application in the study of criminal behavior, explaining how the costs and benefits of crime influence criminal behavior (56, 57). Subsequently, this theory has been widely used in various aspects such as enterprise decision-making regarding production, output, investment, and technological change; individual choices concerning marriage, fertility, and education; personal and household choices related to consumption and savings, public policies and public choices, group and organizational behaviors in sociology, etc. For example, the theory has been employed to explain the relationship between chatbots’ “efficiency-flexibility ambidexterity” and customer usage behavior (58). The results reveal that as privacy concerns diminish and opportunity costs decrease, the positive effect increases. Furthermore, Salhieh (59) have utilized this theory to rationalize users’ switching behavior in mobile services, simulating customers’ service switching behavior and predicting the likelihood of customers switching mobile services. In summary, rational choice behavior is motivated by the individual’s benefit and cost, which are perceived or defined by the individual.

In a similar study of online healthcare platforms, Zhang et al. analyzed how patients’ decisions to choose online services influenced by benefits and costs when faced with two health service provider channels from the perspective of RCT (15). This article thoroughly discussed the influence of benefit factors such as online medical information resources, medical resources, and emotional support, as well as cost factors such as diagnostic errors, information leaks, and expenses of online services. Similarly, in our study, we also consider costs and benefits as factors in determining whether to choose online diagnosis and treatment services. Therefore, we deem the RCT as one of the valuable theoretical frameworks for our research.

### 2.3 Protection motivation theory

PMT, originally introduced by (60), is a theory of health psychology, offering a profound understanding of human reactions to risk. According to this theory, humans are motivated to safeguard themselves by evaluating the potential threat posed by events and assessing their coping abilities, ultimately leading to the adoption of healthy behaviors (61). Threat appraisal, a crucial component of PMT, involves the perceived severity (PS) of an event and perceived vulnerability (PV) which is defined as an individual’s perception of the likelihood of harm when exposed to that event. On the other hand, coping appraisal, including response efficacy (RE) and self-efficacy (SE), refers to the expectation that a patient can eliminate the threat by taking a particular action, alone with the confidence in successfully performing the behavior. Existing literature confirms that when the threat assessment is high, it enhances the protective motive; at the same time, coping appraisal increases the likelihood of individuals adopting effective protective behaviors (62).

Many scholars have utilized this theory to gain insights into individuals’ reactions and coping behaviors towards threats, enabling them to develop more effective behavior promotion strategies. For example, (63) used this theory to analyze the importance of individual preventive measures in the security of computers and the internet in household environments, thereby promoting safe behavior among household users. Hsieh et al. (64) explained, during the COVID-19 pandemic, customers’ perceived level of threat significantly reduced their willingness to hotels. Conversely, self-efficacy significantly increased their willingness to stay. Similarly, Qiao et al. (65) conducted a study examining the protective motives of prospective Korean tourists toward traveling to China during the COVID-19 pandemic. The findings revealed that the threat appraisal and coping appraisal had a significant influence on individuals’ motivations to safeguard themselves, and suggested that media coverage played a moderating role in these relationships. Furthermore, the impact of PMT theory on residents’ hope and fear psychology during the COVID-19 pandemic was discussed, explaining changes in hygiene behavior, restaurant dining behavior, and consumption awareness among individuals under these psychological emotions (66).

In terms of medical health management, PMT is frequently used in disease prevention. For example, Estebsari et al. (67) analyzed the important role of PMT in improving women’s perception and intention rates of breast cancer risk, thus changing their health protection behaviors; Lin and Chang (68) used PMT to explain the intention of Chinese adult smokers to quit smoking, aiming to provide valuable smoking cessation intervention programs from both theoretical and cultural perspectives. Currently, there are relatively few studies focusing on the application of PMT in online healthcare. Zhang et al. (69) analyzed the factors influencing the use of mobile health services (MHS) based on PMT, proving that patients’ perception of disease susceptibility, severity of the disease, response efficacy, and self-efficacy have a positive impact on their intention to use MHS, almost no studies have discussed the influence of external air factors. Therefore, this article assumes that when patients perceive a high potential threat of air pollution, their willingness to self-protect will increase. When individuals perceive the effectiveness of reducing outdoor activities as a protective measure, their intention to seek online medical consultation will increase.

## 3 Research model and hypotheses development

**Figure 1:**
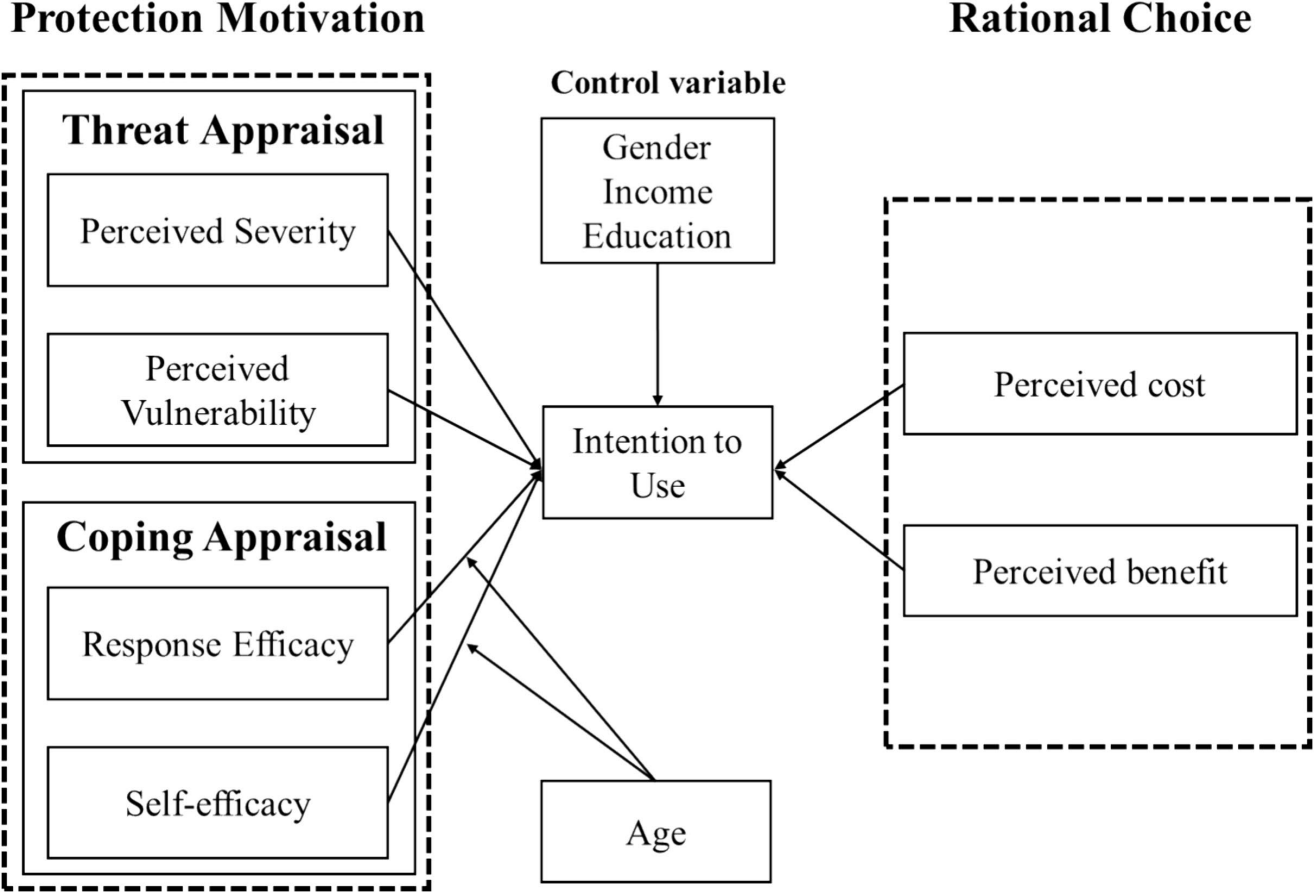
Research model.

To analyze the factors influencing the OHS use intention, we propose an integrated model based on the RCT and the PMT, as shown in Figure 1. On one hand, this model is built upon the RCT framework, focusing on the costs and benefits evaluated by the patient, which are represented by perceived cost (PC) and perceived benefit (PB), respectively. The PMT, on the other hand, emphasizes patients’ threat appraisal and coping appraisal regarding environmental pollution, which include PS, PV, RE, and SE. Notably, we focus on individuals’ perceptions of air pollution, so we did not directly use objective air pollution level data.

### 3.1 Effects of threat appraisal and coping appraisal on OHS use intention

According to the PMT, when air pollution becomes severe, individuals’ perception of a high degree of PS and vulnerability PV can trigger protective motivations, leading to protective behaviors such as avoiding going outdoors (19, 36). In our study, PS refers to individuals’ perceptions of the health hazards posed by air pollution, while PV refers to individuals’ perceived likelihood of experiencing health risks from air pollution. Simultaneously, the PMT has also been used to study individuals’ coping behaviors in the context of health threats. Such studies suggest that high RE and SE can also trigger protective behaviors (65). In this study, RE is defined as confidence in the effectiveness of OHS services in mitigating or preventing health threats from air pollution, while SE represents an individual’s self-assurance in successfully utilizing OHS. Based on these definitions, we propose the following hypotheses:

*H1a: Severity perception from air pollution has a positive and significant impact on people’s intention to use.*

*H1b: Vulnerability perception from air pollution has a positive and significant impact on people’s intention to use.*

*H2a: Response efficacy perception of OHS has a positive and significant impact on people’s intention to use.*

*H2b: Self-efficacy has a positive and significant impact on people’s intention to use.*

### 3.2 Effects of perceived benefits and perceived cost on OHS use intention

According to RCT, when patients are presented with an alternative choice, they will conduct a benefits-costs assessment. The benefits of utilizing OHS include improved health and resolution of health issues (3, 15), as well as providing an online channel to minimize the threat of air pollution (70). Based on this, we hypothesize that patients’ perceptions of benefits will motivate them to seek those benefits by utilizing OHS. Conversely, the perception of costs, arising from concerns about internet hospitals’ inability to cure illnesses, potential interference with treatment, and the high financial burden, is hypothesized to deter patients from utilizing OHS (71–73). This leads to our hypothesis:

*H3a: Perceived benefit is positively associated with OHS use intention.*

*H3b: Perceived cost is negatively associated with OHS use intention.*

### 3.3 Moderate effect of age on OHS use intention

Based on existing research, individuals’ behaviors and perceptions are influenced by age (74). Studies examining the impact of air pollution reveal that age-related differences lead to varying health outcomes (75, 76). Hernández et al. (77) employed age as a moderating variable to discuss changes in the beliefs and behaviors of online shoppers. Moreover, age is a critical personal characteristic in the adoption of new technological services. *N* numerous studies indicate that younger individuals are more likely to adopt new information technologies compared to older individuals (78–80). Recognizing that age significantly influences individuals’ willingness to adopt and effectively utilize OHS (81–83), our study incorporates age as a moderator variable to investigate its modulation effect on the association between coping appraisal and use iteration. Based on this, we propose the following hypothesis:

*H4a: Patients’ age moderates the relationship between RE and the intention to use online medical services.*

*H4b: Patients’ age moderates the relationship between SE and the intention to use online medical services.*

Based on the above analysis, we propose a model of consumer resistance to seafood products within the SOR framework, as shown in Figure 1.

## 4 Method

### 4.1 Data collection

An online questionnaire was developed to evaluate our theoretical model. This questionnaire was designed to ensure that our research subjects have sufficient means to access the Internet and thus have the ability to choose online medical services. We use Questionnaire Star^1^ to push questionnaires to potential people through WeChat channels. When introducing OHS, we primarily focused on diagnostic consultation services provided by platforms such as Haodf and Huayitong. Target participants include residents of China, fluent in Chinese, and over 20 years old, indicating their capability for independent medical decision-making. To motivate serious engagement, participants were offered a random cash incentive upon completion. The survey, conducted between January 14 and 21, 2024, involved 589 participants. The survey period coincided with winter in China, characterized by widespread haze. To maintain questionnaire integrity, selection criteria were applied, including a single response per account and a minimum response time of 300 s to exclude superficial responses. After excluding 47 participants for failing the attention check, 542 remained, yielding an effective response rate of 92.02%.

### 4.2 Measures

This study’s questionnaire comprised three sections. The first section explains the questionnaire’s purpose, upholds ethical standards, and emphasizes three points: (1) encouraging honest responses without right or wrong answers to minimize bias (2). Ensuring anonymity and confidentiality of responses (3). Restricting the use of collected data to academic research, excluding commercial use. To ensure answer reliability, an attention check was integrated into the questionnaire. The questionnaire was drafted in Chinese and was initially completed by four relevant personnel who have long been engaged in online medical service research to ensure translation accuracy. They provided effective feedback information for the improvement of the questionnaire.

The second section of the online questionnaire is the core part, with 21 questionnaire items designed to measure the 7 constructs mentioned in the theoretical model. Each item exclusively measures one construct. All items are sourced from relevant research literature, and we have made moderate adaptations to make them more consistent with our research scenarios. Items were rated on a 7-point Likert scale from (1) strongly disagree to (7) strongly agree. Table 1 shows all constructs, items and reference sources.

**Table 1:** Constructs, items, and references of the measurements.

Construct	Definition and items	References
Perceived severity (PS)	Individuals’ perceptions of the health hazards posed by air pollution	(36, 66, 92, 93)
PS1	Air pollution, comprising sulfur dioxide and carbon monoxide, is detrimental to health	
PS2	Exposure to air pollution may exacerbate my illness	
PS3	Significant health impacts may result from air pollution exposure	
Perceived vulnerability (PV)	Individuals’ perceived likelihood of health risks from air pollution	
PV1	Air pollution has a high probability of harming health	
PV2	Air pollution may worsen illnesses	
PV3	Health is vulnerable to adverse effects from air pollution	
Self-efficacy (SE)	Individuals’ self-assessed ability to use online medical services	
SE1	It is easy for me to use online medical services	
SE2	I am capable of accessing online medical services	
SE3	Online medical services require minimal effort to use	
Response efficacy (RE)	Perceived efficacy of online medical services in mitigating air pollution exposure	
RE1	Reduced exposure to air pollution can protect health	
RE2	Online medical services can prevent the health hazards associated with air pollution exposure	
RE3	Online medical services can reduce air pollution-related health risks	
Perceived benefits (PB)	Perceived benefits of utilizing online medical services	(3, 15, 94)
PB1	Online medical services can improve my health	
PB2	Online medical services can solve my health problems	
PB3	Online medical services can reduce the threat of air pollution to my health	
Perceived costs (PC)	Perceived costs associated with the use of online medical services	
PC1	Using online medical services may not completely cure my disease	
PC2	Utilizing online medical services could potentially affect my disease treatment	
PC3	The cost associated with using online medical services can be significant	
Intention to use (IU)	Willingness to utilize online medical services	(95, 96)
IU1	I plan to use online medical services when air pollution is severe	
IU2	I am inclined to use online medical services during times of serious air pollution	
IU3	To avoid going out for medical care, I would prefer using online medical services when air pollution levels are high	

The third section gathers demographic information such as gender, age, education, marital status, and annual disposable income, categorizing them accordingly (e.g., male = 0, female = 1). Average annual income was categorized based on the 2022 classification for Chinese residents’ average disposable income: low, lower-middle, middle, upper-middle, and high.

### 4.3 Data analysis

We computed descriptive statistics using Python 3.9. To evaluate the fit between measurement model and our theoretical framework, as well as to verify the items’ internal consistency and construct validity, we conducted confirmatory factor analysis (CFA). Using SmartPLS 4.0 software, we applied PLS-SEM, which is noted for its efficacy in estimating complex models with latent constructs (84), to examine the significant relationships among the variables.

## 5 Results

### 5.1 Demographic analysis

This research examined the demographic characteristics of 542 participants to thoroughly understand the sample population’s fundamental attributes. In terms of gender distribution, male and female participants accounted for 44% (239 individuals) and 56% (303 individuals) of the sample, respectively. Age distribution showed that the 20 to 29 age group made up 36% (196 individuals), with the 50 to 59 age group following at 22% (116 individuals). Educational analysis indicated 60% (328 individuals) held university degrees, and 10% (53 individuals) possessed graduate degrees. Marital analysis found that 75% (409 individuals) were married and 25% (133 individuals) were single. Economic analysis revealed the lower-middle income group constituted 25% (134 individuals), and the low-income group 12% (64 individuals), suggesting a balanced income distribution. For further details, refer to Table 2. The demographic data laid a solid foundation for this research, enabling a comprehensive understanding of the sample characteristics. The income distribution’s similarity to that of Chinese residents bolsters the sample’s representativeness, ensuring an accurate portrayal of overall characteristics.

**Table 2:** Respondent characteristics.

Items	Category	Frequency (*n* = 542)	Proportion
Gender			
	Male (0)	239	0.44
	Female (1)	303	0.56
Age			
	20–29 (1)	196	0.36
	30–39 (2)	88	0.16
	40–49 (3)	109	0.2
	50–59 (4)	116	0.22
	60及以上	33	0.06
Educational level			
	Less than high school (1)	161	0.3
	University (2)	328	0.6
	Graduate school (3)	53	0.1
Marital status			
	Single (0)	133	0.25
	Married (1)	409	0.75
Average annual income (¥)			
	Low-income group	64	0.12
	Lower-middle-income group	134	0.25
	Middle-income group	128	0.24
	Upper-middle-income group	127	0.23
	High-income group	89	0.16

### 5.2 Measurement model results

We employed SmartPLS for confirmatory factor analysis (CFA) to assess the reliability and validity of the collected questionnaire data. The reliability of this study was assessed using Cronbach’s *α* (C. A.), composite reliability (CR), and average variance extracted (AVE). All results, presented in Table 3, reveals that the Cronbach’s *α* values for each construct range from 0.812 to 0.848, surpassing the recommended threshold of 0.7 (36), indicating acceptable reliability for each construct. Additionally, all items exhibited factor loadings greater than 0.7, demonstrating strong convergent validity (85). All CR values, utilized for assessing the internal consistency of each construct, exceed the 0.7 threshold, reflecting high consistency and robust reliability (15). Finally, the variance inflation factor (VIF) was used to check for multicollinearity among items. All VIF values were below 3, significantly lower the critical value of 10 (86).

**Table 3:** Measurement scale properties.

Construct	Items	Factor loading	C. A.	CR (rho_a)	CR (rho_c)	AVE	VIF
Perceived vulnerability (PV)	PV1	0.881	0.848	0.848	0.908	0.767	2.078
	PV2	0.873					2.039
	PV3	0.873					2.034
Perceived severity (PS)	PS1	0.849	0.812	0.812	0.889	0.727	1.746
	PS2	0.847					1.752
	PS3	0.861					1.853
Self-efficacy (SE)	SE1	0.87	0.83	0.83	0.898	0.746	1.931
	SE2	0.855					1.82
	SE3	0.866					1.961
Response efficacy (RE)	RE1	0.867	0.839	0.839	0.903	0.757	1.935
	RE2	0.874					1.993
	RE3	0.868					1.994
Perceived benefits (PB)	PB1	0.878	0.835	0.836	0.901	0.752	2.037
	PB2	0.86					1.912
	PB3	0.863					1.891
Perceived costs (PC)	PC1	0.857	0.836	0.837	0.901	0.753	1.84
	PC2	0.885					2.109
	PC3	0.861					1.953
Intention to use (IU)	IU1	0.87	0.828	0.828	0.897	0.744	1.976
	IU2	0.847					1.753
	IU3	0.871					1.993

C. A., Cronbach’s alpha; AVE, average variance extracted; CR, composite reliability; VIF, variance inflation factor.

The AVE values, employed to assess the validity of all constructs, exceed the 0.5 threshold, which indicate strong convergent validity (87). Table 4 presents the discriminant validity analysis results, based on the Fornell–Larcker criterion. Given that the square root of AVE exceeded the inter-construct correlation coefficients, the data demonstrate acceptable discriminant validity (85). To further validation, a cross-loading analysis was conducted, as detailed in Table 5. The cross-loadings for all items significantly exceeded the 0.70 benchmark for their respective constructs (88). Furthermore, loadings within each construct exceeded those across other constructs, reinforcing discriminant validity.

**Table 4:** Discriminant validity analysis with Fornel–Larcker criterion.

Construct	IU	PB	PC	PS	PV	RE	SE
IU	0.863						
PB	0.827	0.867					
PC	−0.828	−0.826	0.868				
PS	0.83	0.83	−0.836	0.852			
PV	0.824	0.822	−0.816	0.829	0.876		
RE	0.841	0.831	−0.83	0.835	0.828	0.87	
SE	0.831	0.846	−0.834	0.833	0.838	0.837	0.864

**Table 5:** Discriminant validity analysis with cross loadings.

	IU	PB	PC	PS	PV	RE	SE
IU1	0.87	0.713	−0.714	0.711	0.727	0.735	0.722
IU2	0.847	0.734	−0.716	0.711	0.693	0.718	0.726
IU3	0.871	0.694	−0.712	0.725	0.713	0.724	0.702
PB1	0.731	0.878	−0.716	0.744	0.721	0.718	0.74
PB2	0.697	0.86	−0.711	0.693	0.702	0.718	0.727
PB3	0.722	0.863	−0.723	0.721	0.714	0.725	0.734
PC1	−0.723	−0.715	0.857	−0.737	−0.707	−0.717	−0.727
PC2	−0.74	−0.726	0.885	−0.739	−0.718	−0.734	−0.732
PC3	−0.69	−0.709	0.861	−0.701	−0.7	−0.708	−0.711
PS1	0.712	0.705	−0.727	0.849	0.701	0.719	0.717
PS2	0.701	0.695	−0.698	0.847	0.7	0.695	0.704
PS3	0.708	0.722	−0.714	0.861	0.718	0.72	0.709
PV1	0.737	0.721	−0.709	0.728	0.881	0.745	0.739
PV2	0.71	0.723	−0.71	0.72	0.873	0.713	0.731
PV3	0.717	0.715	−0.725	0.729	0.873	0.715	0.729
RE1	0.735	0.717	−0.713	0.727	0.706	0.867	0.731
RE2	0.746	0.736	−0.734	0.734	0.743	0.874	0.741
RE3	0.713	0.715	−0.717	0.716	0.71	0.868	0.712
SE1	0.737	0.751	−0.732	0.731	0.755	0.726	0.87
SE2	0.716	0.716	−0.704	0.708	0.713	0.719	0.855
SE3	0.699	0.723	−0.723	0.719	0.701	0.724	0.866

### 5.3 Structural model results

We employed the PLS-SEM algorithm to assess the structural model and quantify variable relationships. The model showed a good fit, achieving a goodness of fit score of 0.779, which exceeds the benchmark of 0.36 (89). Rational choice and protection motivation account for 80.7% of the variance in the intention to use online medical services. Furthermore, all variables’ *Q*^2^ values surpass 0.35, highlighting the model’s strong predictive capability (90). Model fit evaluation metrics are presented in Table 6. These metrics, which aid in preventing model misspecification (91), show a satisfactory fit, with SRMR = 0.047, d_ULS = 0.551, d_G = 0.446, and NFI = 0.862, confirming the analysis’s robustness.

**Table 6:** Recommended and actual values of fit metrics.

Fit indices	Recommended value	Saturated model	Estimated model
SRMR	<0.08	0.036	0.042
d_ULS	<0.95	0.415	0.568
d_G	<0.95	0.425	0.456
NFI	>0.8	0.865	0.860

SRMR, standardized root mean square residual; NFI, normed fit index.

**Figure 2:**
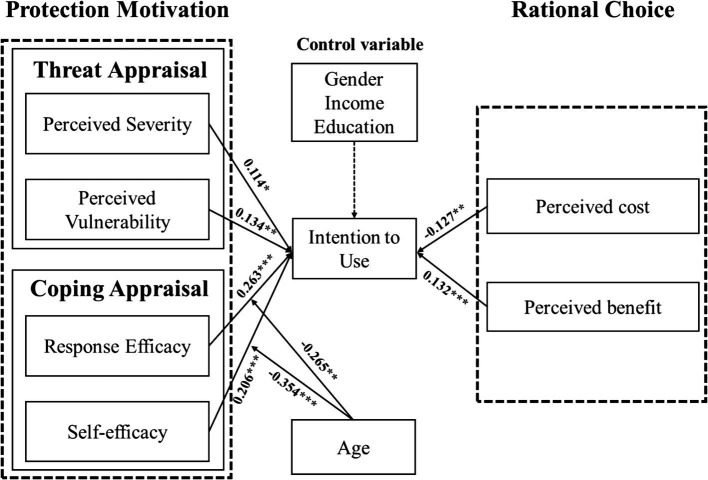
Results of statistical analysis of the proposed conceptual model. Solid line indicate significant relationships and dotted lines indicate insignificant relationships; *p < 0.05, **p < 0.01, ***p < 0.001.

We employed PLS-SEM to test our hypothetical model. As depicted in Table 7 and Figure 2, the direct effects among all variables are illustrated, and all hypotheses were supported and validated. Initially, from the perspective of protection motivation, perceived air pollution threats were found to enhance the intention to use online medical services. Specifically, PS (*β* = 0.114, *p* < 0.05) and PV (*β* = 0.134, *p* < 0.01) positively influenced this intention, validating H1a and H1b. Furthermore, the results shown that evaluating the effectiveness of using online medical services to counter air pollution threats boost the intention to use these services. RE (*β* = 0.263, *p* < 0.001) and SE (*β* = 0.206, *p* < 0.001) have a significant positive effect, supporting H2a and H2b. According to rational choice theory, PB increases the intention to use online medical services, while PC decreases this intention, with *β* = 0.132 (*p* < 0.001) and *β* = −0.127 (*p* < 0.01), respectively, thus confirming H3a and H3b.

**Table 7:** Direct path analysis.

Hypotheses	Path coefficient	Standard deviation	Support
H1a: PV → IU	0.134**	0.043	Y
H1b: PS → IU	0.114*	0.046	Y
H2a: RE → IU	0.263***	0.044	Y
H2b: SE → IU	0.206***	0.052	Y
H3a: PB → IU	0.132***	0.041	Y
H3b: PC → IU	−0.127**	0.043	Y

*p < 0.05, **p < 0.01, and ***p < 0.001.

**Figure 3:**
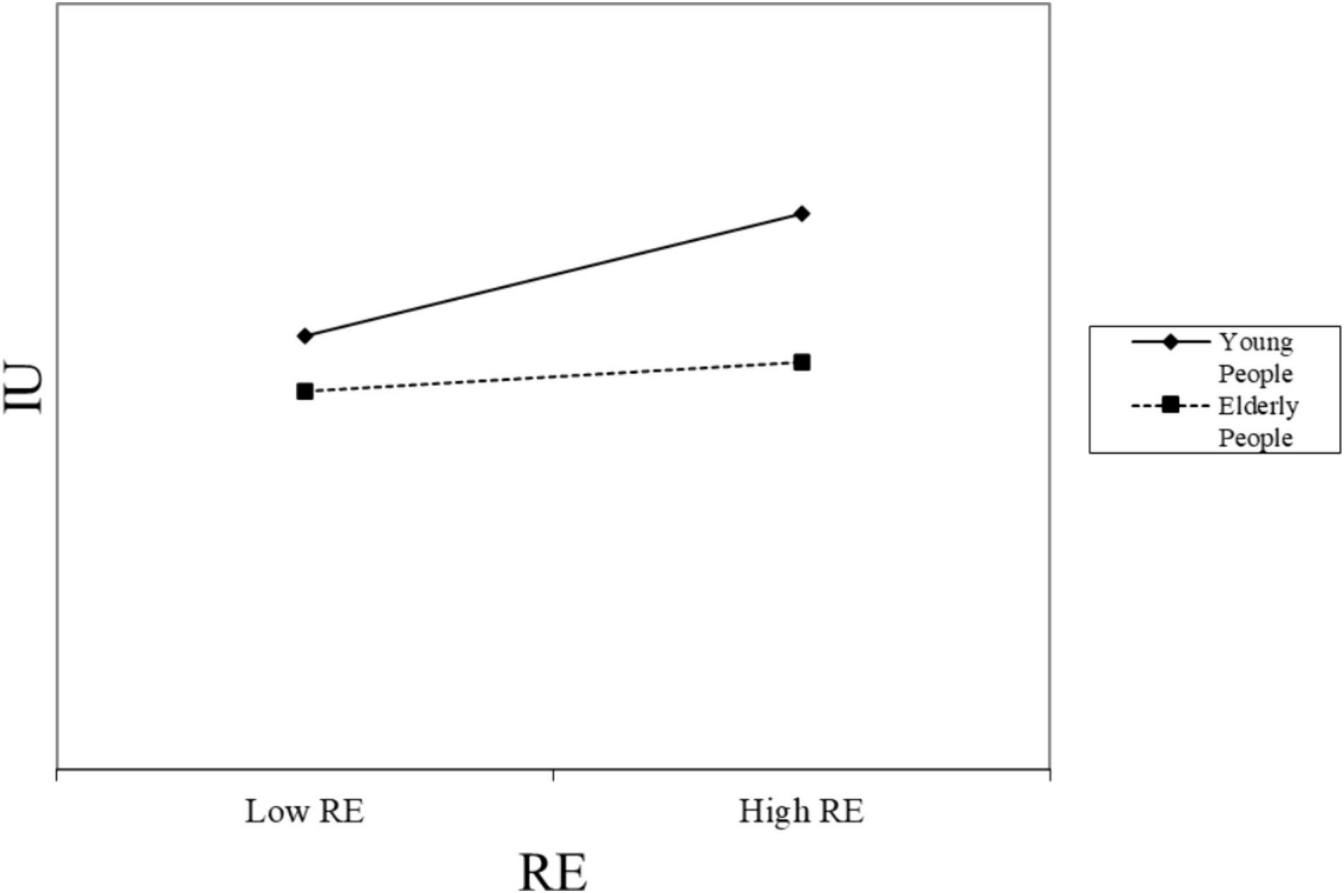
Moderating role of gender on RE.

**Figure 4:**
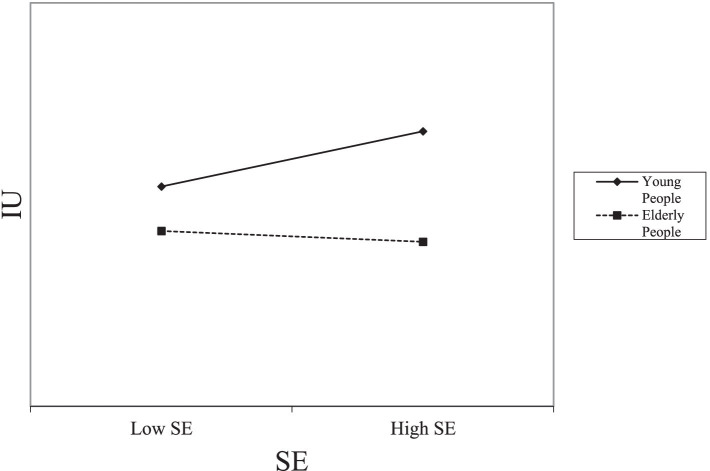
Moderating role of gender on SE.

Additionally, we employed PLS-SEM in SmartPLS to test the moderating effect of age on the relationship between coping appraisal and the intention to use online medical services. A significant interaction was observed between age and coping appraisal, which is presented in Table 8 and Figures 3, 4. Specifically, the interaction between age and RE (*β* = −0.265, *p* < 0.01), as well as between age and SE (*β* = −0.354, *p* < 0.001), were significantly related to the intention to use online medical services. These results suggest that the positive impact of coping appraisal on the intention to use decreases with age. Figures 3, 4 illustrate the moderating effect of age, respectively.

**Table 8:** Moderating effect analysis.

Hypotheses	COEF	STD	*T* value	*p*
Age → IU	0.310	0.082	3.776	0.000
RE → IU	0.263	0.044	6.005	0.000
SE → IU	0.206	0.052	3.972	0.000
H4a: Age × RE → IU	−0.265	0.097	2.764	0.006
H4b: Age × SE → IU	−0.354	0.102	3.471	0.001

## 6 Discussion and conclusions

OHS is rapidly expanding, offering patients an innovative and convenient approach to medical consultations. While researchers have thoroughly examined patient interest in using OHS, the influence of environmental factors like air pollution has not been extensively explored. This study combines the protection motivation theory and rational choice theory to assess how air pollution, as an external threat factor, influences patients’ willingness to use OHS. To validate our model, we utilized cross-sectional data from China. The results indicate that air pollution increases patients’ self-protection awareness, thereby boosting their inclination to use OHS. From the perspective of rational choice, perceived benefits of OHS enhance its usage, whereas perceived drawbacks deter it.

In this study, our results indicate that the threat of air pollution increases the propensity of people to use OHS (H1a, H1b). Specifically, perceived severity (PS) and perceived vulnerability (PV) have a positive direct effect on the willingness to use OHS. This suggests that users perceive air pollution as a serious threat to their health, thereby promoting the use of OHS. As suggested in (62), the drive for self-protection leads individuals to adopt advantageous actions. Research in the retail and tourism industries demonstrates that significant air pollution activates individuals’ protective instincts, leading to a decrease in outdoor activities (19, 36). Our results corroborate this view, indicating that threat evaluation foster self-protective actions in individuals.

Besides threat appraisal, our research also found that coping appraisal is a significant positive predictor of users’ willingness to use OHS. Specifically, response efficacy (RE) and self-efficacy (SE) significantly promote the willingness to use OHS (H2a, H2b). This indicates that when users believe that using OHS can effectively reduce health risks (high response efficacy) and have confidence in their ability to use these services (high self-efficacy), they are more likely to choose OHS. This further emphasizes the importance of enhancing users’ trust and competence in using OHS to increase its adoption and usage. Furthermore, it is noteworthy that we found the positive influence of coping appraisal on the inclination to use OHS decreases with increasing age. Guo et al. (92) reached a similar conclusion regarding the adoption of mobile health services. One potential explanation is the varied acceptance of OHS across different age groups. According to this research (80), younger individuals are more likely to embrace digital technologies, while older individuals tend to demonstrate lower levels of acceptance, which is consistent with our study’s findings. Compared to their younger counterparts, older users exhibit a less robust coping appraisal of OHS. Therefore, as age advances, the positive effect of coping appraisal on the readiness to utilize OHS diminishes.

Our findings further confirm the critical role of perceived benefits (PB) and perceived costs (PC) in promoting the use of OHS (H3a, H3b). Beyond self-protection motivation, rational choice plays a crucial role in understanding individual decision-making. RCT posits that the evaluation of benefits encourages decision-making behaviors, while the consideration of costs acts as a deterrent (3, 72). Zhang et al. (15) explored how perceived benefits and costs influence the willingness to use online medical services. Our results align with this perspective, showing that the perception of benefits encourages the use of OHS, while concerns about costs discourage adoption. This may be because PB can significantly enhance users’ acceptance and willingness to use OHS, as they believe it brings tangible health benefits and convenient medical services. In contrast, PC significantly reduces users’ willingness to use OHS due to concerns about potential financial burden, time costs, and inconveniences during use.

This study addresses several gaps in the existing literature by examining how air pollution, as an environmental factor, influences patients’ willingness to use OHS. This research provides crucial theoretical foundations for the promotion of OHS. Additionally, this study integrates PMT and RCT to investigate the determinants of patients’ willingness to engage in OHS under the threat of air pollution. By combining PMT’s self-protection motivation framework with RCT’s cost-benefit evaluation model, we can comprehensively understand patients’ decision-making processes when facing environmental health threats. Furthermore, our findings reveal that age moderates the impact of coping appraisal on the propensity to use OHS. Specifically, while coping appraisal under the threat of air pollution generally enhances patients’ willingness to use OHS, this positive effect diminishes significantly with increasing age. This finding underscores the importance of considering age-related factors in technology acceptance when promoting OHS, and highlights the need for tailored strategies for different age groups to maximize the adoption of OHS.

In practical terms, our research provides valuable insights for the promotion and enhancement of online healthcare services (OHS). Firstly, we offer guidance for healthcare providers by analyzing the factors influencing patients’ willingness to adopt OHS amidst air pollution threats, a critical yet underexplored area in current research. Our findings suggest that during periods of air pollution, managers can optimize the allocation of online and offline medical resources to better accommodate fluctuating patient demand, ensuring efficient service delivery and resource utilization. Furthermore, our study highlights the differences in OHS adoption across various age groups, particularly in terms of occupational health and safety considerations. This understanding is crucial for tailoring OHS products to meet the diverse needs of various age demographics. For instance, simplifying system usability and enhancing accessibility can significantly increase the adoption rates among older individuals. By addressing these age-related differences and preferences, healthcare providers can develop more inclusive and user-friendly OHS platforms, ultimately improving the overall patient experience and encouraging broader adoption across all age groups.

Although our study contributes significantly to the existing literature, it has certain limitations. Firstly, our sample size is relatively small. Despite passing reliability and validity tests, a larger sample size could further validate the robustness of our conclusions. Secondly, our research primarily focuses on diagnostic consultation services, and our findings may not be fully applicable to other types of services, such as medical prescriptions or knowledge sharing. Additionally, our study is confined to the Chinese context, meaning that cultural differences could potentially influence our conclusions. This limitation highlights the need for future research in diverse cultural settings to generalize our findings. Lastly, our study relies on subjective survey data, inherently carries a degree of bias. To address these limitations, we are actively seeking more objective data from a major Chinese hospital; and negotiations are currently ongoing.

## Data Availability

The raw data supporting the conclusions of this article will be made available by the authors, without undue reservation.

## Footnotes

^1^https://www.wjx.cn/
